# Use of goat interleukin-6, cortisol, and some biomarkers to evaluate clinical suitability of two routes of ascorbic acid administration in transportation stress

**DOI:** 10.14202/vetworld.2018.674-680

**Published:** 2018-05-22

**Authors:** K. T. Biobaku, T. O. Omobowale, Ahmed O. Akeem, A. Aremu, N. Okwelum, A. S. Adah

**Affiliations:** 1 Department of Veterinary Pharmacology and Toxicology, University of Ilorin, Nigeria; 2 Department of Veterinary Medicine, University of Ibadan, Nigeria; 3 Department of Veterinary Physiology and Biochemistry, University of Ilorin, Nigeria; 4 Department of Veterinary Microbiology, University of Ilorin, Nigeria; 5 Institute of Food Security, Environmental Resources and Agricultural Research, IFSERAR, Federal University of Agriculture, Abeokuta, Nigeria

**Keywords:** ascorbic acid, intramuscular, oral, Kalahari goats, stress

## Abstract

**Aim:**

The study determined the effect of ascorbic acid (administered orally and intramuscularly) in short-term transportation stress.

**Materials and Methods:**

Twenty-four apparently healthy Kalahari goats were grouped into four groups (A, B, C, and D) of 6 animals each: Group A - untreated and unexposed to stress; Group B - treated with 200 mg/kg Vitamin C orally and exposed to 2 h transportation stress; Group C - treated with Vitamin C 200 mg/kg intramuscularly and exposed to 2 h transportation stress; and Group D - untreated and exposed to 2 h transportation stress. The animals were stocked using standards stipulated by the Nigerian Animal Disease Control Act and transported at 40 km/h. Cortisol and interleukin-6 (IL-6) were assayed using quantitative sandwich ELISA. Classical stress hematological parameters and antioxidative stress markers such as glutathione s-transferase, superoxide dismutase, and malondialdehyde were determined. Heart rate variability (HRV) was also assessed.

**Results:**

The route of ascorbic acid administration did not influence the expression of IL-6, and changes in cortisol surge, antioxidative stress markers, and other hematological parameters in Kalahari goats though Group C goats showed higher HRV values (p<0.05) than others. This gives credence to the enhanced cardiac responsiveness and stress survivability in Kalahari goats.

**Conclusion:**

Both routes could be used in the administration of ascorbic acid. Kalahari goats exposed to short-term stress; however, the intramuscular route had better heart variability and thus improved the survivability of the animals.

## Introduction

The pharmacokinetics of ascorbic acid (vitamin C) is important in animals. Ascorbic acid has antioxidative functions due to its redox properties [1]. It improves cell-mediated immunity and mops up oxygen radicals. It has the enzyme gulonolactone oxidase responsible for the conversion of gulonic acid to gulanolactone in ascorbic acid endogenous synthesis [2]. Ascorbic acid is important in increasing the resistance against respiratory diseases [1,3]. The deficiency of ascorbic acid could manifest as scurvy in animals while calves can show scurvy like skin lesions [4].

Calves and lambs depend on the high level of ascorbic acid in colostrum and milk from the dam [4]. Young ruminants require more ascorbic acid during cold stress, and plasma concentration should be high to confer immunity in the animals. It helps to prevent neonatal calve diarrhea and scours [5]. Supplementing young lambs and calves to achieve high plasma or serum concentration is easier since they are able to utilize exogenous oral ascorbic acid. In adult ruminants, supplementation with ascorbic acid is done intramuscularly to avoid its destruction in the rumen. Therefore, it will be difficult to achieve a high serum or plasma concentration. Ascorbic acid is a dietary requirement in primates, guinea pig, rabbits, and various exotic species such as mink and ferret. Ascorbic acid could be a safe and cheap means to ameliorate the effect of transportation stress in food animals such as goats.

Road transportation is an inevitable animal husbandry practice in sub-Saharan Africa [6]. Animal welfare in Nigeria is neglected, and this greatly affects animal production. Food animal transportation to slaughterhouses across the country is done in substandard ways. Most slaughterhouses lack functional designated lairages for resting animals transported before slaughter while laws enacted to regulate these activities are usually lax and unenforced. This affects the quality of meat derived from such animals slaughtered for consumption. More so, animal transportation for slaughter purpose in Nigeria is associated with huge economic loss [7]. Vitamin C supplementation could be of economic importance in the meat industry through stress alleviation in transported animals. Stress in animals compromises health and quality of products [8-10]. Meat from stressed animals could cause ill health in humans. Vitamin C to some extent possesses immunomodulatory effect in goats at transportation [10]. It improves electrolyte deregulation and some biochemical parameters as well as prevents muscular soreness in long-term transportation [10,11]. Supplementation with ascorbic acid improved temperature in pigs after short-term transportation [6,12].

In most previous studies on local breeds of animals, ascorbic acid was administered through the oral route [6,9,10,13]. The ability to use other parenteral routes to attenuate stress in animals has not been assessed. There is a tendency that high bioavailability could be achieved using other parenteral routes of administration of ascorbic acid other than the oral route. The oral route was speculated to be unsuitable in ruminants because of ascorbic acid inactivation by the ruminant microflora. It is also difficult to administer ascorbic acid supplement in other species such as swine with the precise dose ensured. The clinico-pharmacological assessment of other parenteral routes used in the administration of ascorbic acid is paramount to enhance effective administration for the rational alleviation of stress. Therefore, the use of oral administration of drug versus the parenteral routes might be readdressed using more sensitive stress assessment markers (not currently used in Nigeria) to ensure the efficacy of the administered ascorbic acid, especially in stress control in ruminants.

Consuming meats derived from stressed animals is unsafe. This can cause impotence in males and varying reproductive cycle anomalies in the females. Epinephrine, norepinephrine, cortisol, and other biochemical stress markers might induce the onset of fluid exudation from meat causing the dark firm dry syndrome [14]. This is detrimental to dynamics of steroidal hormones in humans and could be genotoxic. There is a dearth of information on the effectiveness of Vitamin C administration by parenteral routes alongside the oral route while using sensitive stress markers (for instance, plasma cortisol and interleukin-6 [IL-6]) as stress determinants in the African breed, the Kalahari goats. The study compared the oral and intramuscular routes of ascorbic acid administration in the effective alleviation of short-term transportation stress in Kalahari goats. The level of plasma cortisol and IL-6, hematological stress, and anti-oxidative stress markers such as glutathione s-transferase (GST), superoxide dismutase (SOD), and malondialdehyde (MDA) were evaluated. The heart rate variability (HRV) was also assessed.

## Materials and Methods

### Ethical approval

Ethical consent was obtained from the Institute of Food Security, Environmental Resources and Agricultural Research (IFSERAR), Federal University of Agriculture, Abeokuta, Nigeria with approval code number FUNAB-IFSERAR/APP/2016/002.

### Experimental animals

A total of 24 Kalahari goats of both the sexes (age range: 1-2 years) were used in the experiment. The animals were managed under the intensive system at the animal unit of the Federal University of Agriculture, Abeokuta, Nigeria. The animals were treated prophylactically using albenzole^®^ (Agbara Drug Company, Lagos), 7.5 mg/kg, and penstreptomycin^®^ (Kepro, Holland), 20/25 mg/ml (1 ml/25 kg).

### Experimental design

The 24 Kalahari goats used were randomly grouped into four clusters. Each group had 6 goats.

Group A: Kalahari goats that were rested (unstressed) and untreated - negative control

Group B: Kalahari goats administered with 200 mg/kg of ascorbic acid orally and exposed to 2 h transportation stress

Group C: Kalahari goats administered with 200 mg/kg of ascorbic acid intramuscularly and exposed to 2 h transportation stress

Group D: Kalahari goats exposed to 2 h transportation stress only - positive control

### Loading of animals and transportation stress induction

The internal floor of the truck was cushioned, using sand and sorghum leaves designed specifically to avoid contact with urine and feces during the journey. This also helps to prevent sliding and injury. The floor of the trucks’ dimension and spacing per goat during loading was considered based on the Nigerian Animal Disease Control Act of 1988 as adopted from the International Animal Welfare laws. These goats are highly domesticated and are used to physically handle without been excited. All the Kalahari goats except group A were subjected to an experimental journey in a truck transported at an average speed of 40 km/h as stipulated in the Animal Disease Control Act of 1988 of Nigeria. The goats were transported for 2 h in an open lorry to induce stress.

After stress induction for 2 h, the goats (Groups A, B, C, and D) were assessed for HRV. Both the acoustic jelly and clips were placed using the standard procedure. The goats were allowed to stand while clips were attached as previously described [15,16]. The R-R intervals were measured using an EDAN^®^ - VE 1010 digital veterinary electrocardiographic machine. The HRV was analyzed using a computer programmed beat-beat variation in R-R interval.

### Blood sample collection

After ensuring proper restraint, a 21-gauge needle and syringe was used for blood collection through the jugular vein and sequel to swabbing with methylated spirit and cotton wool to ensure asepsis. 5 ml of blood were collected in separate bottles containing lithium heparin and EDTA for biochemical and hematological analyses, respectively.

### Determination of cortisol and IL-6

Plasma cortisol and IL-6 concentrations were determined using commercial kits (Bioresource, USA; Cat. No. MBS263105 for plasma cortisol and Cat. No. MBS025544 for IL-6). Double ELISA antibody sandwich technique was used for the determination of cortisol in this study. This procedure is characterized by testing antigen with more than two valences.

### Hematological analysis

Blood samples were analyzed as previously described [17]. The parameters determined were red blood cell count (RBC), packed cell volume (PCV), and mean corpuscular hemoglobin concentration (MCHC). White blood cell (WBC) counts were assessed using standard method [17,18].

### Determination of antioxidative enzymes

The GST, SOD, and MDA were assayed using standard methods [19,20] as explained in earlier reports.

### Statistical analysis

The data were subjected to statistical analysis by T-tests and ANOVA to test mean significance between and among groups. p<0.05 was considered to be statistically significant. All analyses were performed using SPSS, version-18. Data obtained from HRV were analyzed as the square root of mean of the sum of squares of difference between successive interbeat intervals of successive heart beats for 5 min.

## Results

### Effects of oral and intramuscular administration of ascorbic acid on IL-6, cortisol, and some other biomarkers in Kalahari goats exposed to 2 h transportation stress

There were no significant differences in cortisol, IL-6, and WBC count in Kalahari goats exposed to 2 h transportation stress (Table-1). There was a significant (p<0.05) higher values of neutrophils (37.83%) in Group D (positive control) as compared to Group A (negative control, 30.17%). There were no significantly different values of neutrophils in Groups B (34.33%) and C (34.67%). However, there were significantly (p<0.05) higher lymphocytes (68.50%) in goats in Group A when compared to the lymphocytes value (61.33%) of Group D goats. No significant lymphocyte values were noted among Groups A (68.50%), B (65.00%), and C (64.50%). Similarly, Kalahari goats in the positive control group had a significantly higher N:L ratio (0.62) compared with other three groups though no significant difference was observed in the N:L ratio between Groups B and C.

**Table-1:** Effects of oral and intramuscular administration of ascorbic acid on stress markers.

Stress determinant	Parameter	Groups				SEM	p-value

		A	B	C	D		
IL-6/cortisol/stress biomarkers	Cortisol (ng/ml)	259.73	254.13	258.50	253.22	4.00	0.06
	IL-6 (Pg/ml)	35.80	32.17	32.33	32.83	2.47	0.70
	WBC×10^9^/L	7.83	10.43	13.85	9.53	2.45	0.39
	Neutrophils (%)	30.17^b^	34.33^ab^	34.67^ab^	37.83^a^	1.70	0.04
	Lymphocytes (%)	68.50^a^	65.00^ab^	64.50^ab^	61.33^b^	1.50	0.03
	N:L	0.43^b^	0.53^ab^	0.53^ab^	0.62^a^	0.04	0.03
Antioxidative markers	GST (µ/ml)	1.26^a^	1.25^a^	1.24^a^	1.12^b^	0.05	0.05
	SOD (Units)	33.44	33.21	33.25	30.25	1.9	0.78
	MDA (µ m/ml)	2.17	2.47	2.40	2.18	1.92	0.38
Erythrocytes indices	PCV (%)	28.67^a^	21.83^c^	24.0^b^	24.83^b^	1.15	0.04
	RBC×10^12^/L	12.7	13.70	14.13	14.65	0.84	0.40
	Hb (g/dl)	7.28	7.75	8.08	8.48	0.59	0.54
	MCHC	34.60	33.45	33.5	34.17	0.41	0.19

Means bearing different superscripts abc along the same row differ significantly (p<0.05). SEM=Standard error of mean, IL-6=Interleukin-6, WBC=White blood cell, GST=Gluthatione s-transferase, SOD=Superoxide dismutase, MDA=Malonyldialdehye, PCV=Packed cell volume, RBC=Red blood cell count, MCHC=Mean corpuscular hemoglobin concentration, Hb=Hemoglobin

### Effects of oral and intramuscular administration of ascorbic acid on some antioxidative markers in Kalahari goats exposed to 2 h transportation stress

The effect of oral and intramuscular administration ascorbic acid on antioxidative enzymes in Kalahari goats exposed to 2 h transportation stress is presented in Table-1. For SOD and MDA, no significant differences were found between the test groups and the controls. The GST (1.12±0.54 μ/ml) was significantly lower (p<0.05) in Group D goats when compared to others (Groups A [1.26±0.04 μ/ml], B [1.25±0.54 μ/ml], and C [1.24±0.29 μ/ml]).

### Effects of oral and intramuscularly administered ascorbic acid in Kalahari goats exposed to 2 h transportation stress on erythrocytes and erythrocytes indices

Table-1 shows the effects of ascorbic acid on erythrocytes and erythrocytes indices in the Kalahari goats exposed to 2 h transportation stress. The PCV values of goats in Group A were significantly (p<0.05) higher (28.67%) than in other groups. No significant differences were found in other erythrocytes indices (RBC, Hb, and MCHC) among goats in Groups B, C, and D. Kalahari goats in Group D had insignificant higher values than the treated Groups B and C.

### Effects of the route of ascorbic acid administration on HRV in Kalahari goats

**Figure-1:**
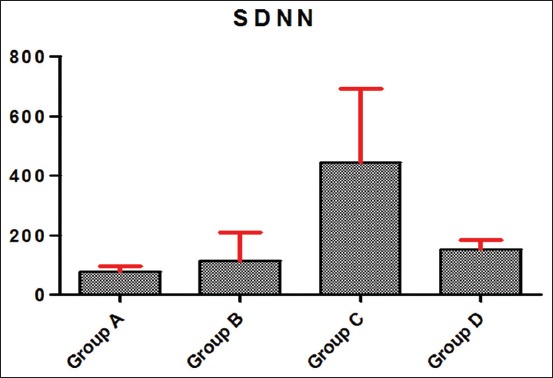
Standard deviation of normal-to-normal interval (standard deviation of interbeat interval). X-axis of graph represents test groups, and Y-axis represents heart rate variability in mean squares. Group A: Kalahari goats that were rested (unstressed) and untreated (negative control); Group B: Kalahari goats administered with 200 mg/kg of ascorbic acid orally and exposed to 2 h transportation stress; Group C: Kalahari goats administered with 200 mg/kg of ascorbic acid intramuscularly and exposed to 2 h transportation stress; Group D: Kalahari goats exposed to 2 h transportation stress only (positive control).

**Figure-2:**
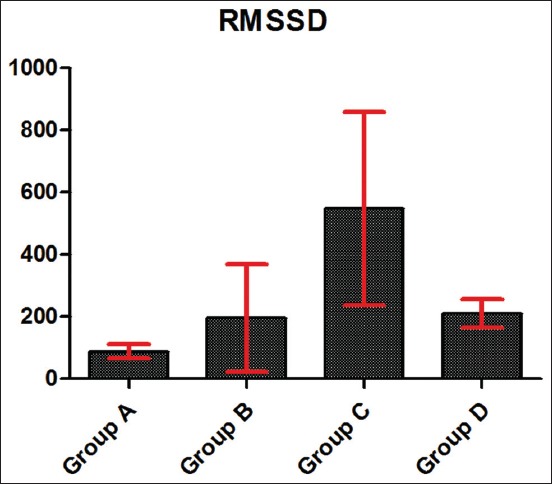
Square root of mean of the sum of squares of differences between interbeat intervals in Kalahari goats exposed to 2 h transportation stress. X-axis of graph represents test groups, and Y-axis represents heart rate variability in mean squares. Group A: Unexposed and Untreated Kalahari goats; Group B: Kalahari goats treated with 200 mg/kg ascorbic acid orally and exposed to transportation stress; Group C: Kalahari goats treated with 200 mg/kg ascorbic acid intramuscularly and exposed to transportation stress; Group D: Untreated Kalahari goats but exposed to transportation stress.

The HRV in Kalahari goats as the standard deviation of interbeat interval and the square root of mean of the sum of square of differences between interbeat intervals in Kalahari goats exposed to 2 h transportation stress are presented in Figures-1 and 2. Kalahari goats in Group C had a significantly (p<0.05) higher HRV than other Groups B, C, and D. Kalahari goats in Group A had the lowest HRV.

## Discussion

We found that the route of ascorbic acid administration did not influence the expression of IL-6, changes in cortisol surge, antioxidative stress markers, and other hematological parameters in Kalahari goats. However, some results observed were interesting, providing better insights in the understanding of the stress dynamics in transported Kalahari goats. There is a dearth of information on expression of IL-6 in Kalahari goats, and this could be peculiar to the hardiness of this breed of goat. Furthermore, the non-expression of IL-6 in these goats might be associated with the distribution of soluble IL-6 receptor, gp130R, in these goats as well as other cytokines such as IL-1α, IL-1β, and TNF1. These can influence the upregulation and downregulation of IL-6 in the Kalahari goats [21,22].

This breed of goats manifested a unique feature - no changes in cortisol surge when compared to the unexposed and untreated control group. This study, unlike an earlier report [19], showed no significant changes in the cortisol level despite the induction of the 2 h transportation stress in these ruminants. There is a high chance that cortisol surge is species dependent as Ajadi *et al*. [19] made use of a different experimental species (bovine). The role of ascorbic acid in the suppression of stress and cortisol surge has been suggested [6,13,19]. Although small ruminants were used in a study [10,14], the difference in the species in relation to the type of stress in which the animals were subjected could account for the difference in the experimental findings. The sensitivity to stress which varies from species to species and even breed of the same species might be associated with a pharmacogenetic factor to variance in the effect of ascorbic acid on stress subjected animals. The route of administration of the supplemental ascorbic acid was found not to significantly influence the surge of cortisol in this study although the use intramuscular route was speculated to increase the cortisol level in the experimental animals [20]. These goats might already be adapted to intensive care and this route of drug administration during routine treatments. This, coupled with the high threshold for pain and level of domestication of this breed of goat, might account for the indifference in cortisol surges in the respective experimental groups in this study. The explanation for the insignificant changes in cortisol could be neuroendocrine, psychosomatic, behavioral, and inherent qualities of this breed of goats.

Our results of hematological readings of stress in the transported Kalahari goats were obvious. The neutrophil and lymphocyte percentages in the systemic circulation of the Kalahari goats reflected the transportation stress, especially in the exposed and untreated group. The two groups that were treated with ascorbic acid showed relatively lower percentage neutrophil values when compared to exposed and untreated. This, therefore, suggests that the route of administration of ascorbic acid did not influence the dynamics of neutrophils percentage. This is, however, different from a previous study which showed that ascorbic acid bioavailability was influenced by formulation, vehicle transportation of drug, and route of administration [5] though species and method of analysis used were dissimilar to that of this study.

The lymphocyte percentages in the same vein were improved on by the ameliorative ascorbic acid when compared to the stressed and untreated Kalahari goats. The route of administration did not influence the lymphocyte percentages in the systemic circulation, but rather the two treatment groups improved hemogram toward the values in the unstressed control group. The neutrophils-lymphocytes ratio is among the parameters of assessment of stress [6,9,13], and in this study, the ascorbic acid decreased the neutrophils-lymphocytes ratio significantly. This provides evidence that ascorbic acids are capable of alleviating transportation-induced stress in Kalahari goats, although the routes of administration showed no significant differences in their extents of decreasing of the neutrophils- lymphocytes ratio.

In this study, PCV was higher in Kalahari goat unexposed to transportation stress when compared to the exposed. This difference is due to the aided stability of the erythrocyte cell membrane of the unexposed goats which prevented hemolysis. There is high likelihood hemolysis of erythrocytes as a result of reactive oxygen species production as a result of stress in the goats exposed to transportation stress. It was, however, suggested by Adenkola *et al*. [23] that ascorbic acid scavenges the reactive oxygen species and thus offers protection to the blood cells. Our findings showed that the exposed and untreated goats also manifested a relatively higher PCV comparable to the group administered with ascorbic acid at 200 mg/kg intramuscularly. This cannot be fully explained, and no categorical statement could be made, but we suggest that the apparent hardy nature of Kalahari breed might be associated with these findings.

The antioxidative stress markers assayed in this study also support that these goats can withstand stress. The 2 h transportation stress induced was insufficient to cause a marked change in the antioxidative enzymes. The unexposed and untreated only manifested a significant decrease in the GST. The lower level of GST in the exposed and untreated group is suggested to be associated with the individual endogenous, cellular antioxidative components of tissues whose concentration would have decreased due to the outweighing effect of the pro-oxidants produced, thus affecting the sum total concentration. The antioxidative enzyme tends to counteract the effect of reactive oxygen and nitrogen radicals produced and protect the cellular macromolecular component. The other antioxidative enzymes, however, were not significantly affected by the induced stress. These goats might have a stable pro-oxidative-antioxidative enzyme ratio to counteract the stress by virtue of inherent genetic makeup. It could be speculated that Kalahari goat breed has a relatively higher plasma ascorbate and other cellular protective mechanisms that could endogenously assist the stability of pro-oxidative-antioxidative ratio.

The HRV in the Kalahari goats used in this study showed that the exposed and (intramuscularly) treated group had higher values compared to others. Administration of the ascorbic acid intramuscularly might have neuroendocrine effects, thus impacting on the HRV of the goats. This might be due to the effect on autacoids, which could be associated with WBC, leukotrienes, and other sensory receptors. The parasympathetic and sympathetic nervous system might be most probably modulated the vagal impulse. The histaminergic, 5-HT, and other receptors could be associated with the change in the rhythmicity of the heart as influenced by the intramuscular route of Vitamin C administration. This non-invasive marker of stress in Kalahari goats might explain the fact that neurohormonal modulation of cardiac function might be influenced by the route of administration of ascorbic acid by prompting the particular concentration of the supplement ascorbic to alleviate stress. The vagal and sympathetic modulation might also be prompted by the ascorbic acid through its indirect involvement in neurotransmission influencing the heart rhythm. Therefore, the heart rate might have responded to cardiac impulse due to instantaneous electrical propagation in the myocytes induced by responses caused by emotional and psychic factors. There are, however, scientific evidence of ascorbic acid involved in ionic sparing in muscles [11]. This can influence the depolarization and repolarization cycle involved in the modulation of the cardiac cycle. Excitability of the animals might also influence the cardiac responsiveness and thus affect the electrical activity of the heart through neurohormonal involvement and changes in contraction, cardiac filling, and stroke volume instantaneous responsiveness to the transportation stress. The HRV in the exposed and treated group of Kalahari goats, unlike other test groups, suggests better survivability. This indicates adaptation of the animals’ emotional, mental, and social behavior after the induction of 2 h stress of transportation. The assumption could also be made that injecting the animals might cause a higher surge of catecholamines and more cardiac stimulation that could account for the prompt changes in the cardiac cycle and interbeat interval and thus the heart variability.

Explanation from the perspective of the vagal tone as an indicator of stress is that direct influence on the sympathetic by activation or blocking may not simply affect the HRV [24,25]. In the groups administered with 200 mg/kg ascorbic acid, the high HRV observed gives an insight of the response of the autonomic response to the 2 h stress, stress reaction, and attempt of the animal’s body to restore homeostasis as a prerequisite to normal visceral function. These findings could be used to monitor physiological response level to stress and a higher tendency for survivability and thus can be used as an index of response and resilience to stress in Kalahari goats. Furthermore, ascorbic acid administered intramuscularly improved the vagal tone better in the experimental goats. This explains the effect of ascorbic acid on autonomic state of the animals and its potential for the restoration of homeostasis. High vagal tone and sensitivity had been associated with adaptation and resistance to adverse conditions such as the stress of transportation [15]. Conversely, low sensitivity might indicate high vulnerability and poor recovery from environmental stress. That high vagal tone in newborn humans was related to better mental, motor, and social abilities [26].

## Conclusion

Both oral and intramuscular routes could be used in the administration of ascorbic acid Kalahari goats exposed to short-term stress; however, the intramuscular route had better heart variability and thus improved the survivability of the animals. Since the exposed and untreated group shows similar HRV level with the goat group exposed and treated with ascorbic acid orally, this further confirms that the Kalahari goat breed has a good stress response ability even with limited supplementation with ascorbic acid. Thus, we recommend this breed be domesticated for production purpose.

## Authors’ Contributions

KTB and AOA conceptualized and designed the study, wrote the protocol, and wrote the first draft of the manuscript. TOO was the consultant and mentor. AA, ASA, and NO contributed in the literature search, data analyses, and interpretation of results. All authors read and approved the final manuscript.
